# Factors Influencing Telemedicine Adoption Among Health Care Professionals: Qualitative Interview Study

**DOI:** 10.2196/54777

**Published:** 2025-01-27

**Authors:** Fiona Schürmann, Daniel Westmattelmann, Gerhard Schewe

**Affiliations:** 1 Center for Management University of Münster Münster Germany

**Keywords:** trust referents, multidimensional risk, benefits, transparency, technology adoption, telemedicine, extended valence framework

## Abstract

**Background:**

Telemedicine is transforming health care by enabling remote diagnosis, consultation, and treatment. Despite rapid adoption during the COVID-19 pandemic, telemedicine uptake among health care professionals (HCPs) remains inconsistent due to perceived risks and lack of tailored policies. Existing studies focus on patient perspectives or general adoption factors, neglecting the complex interplay of contextual variables and trust constructs influencing HCPs’ telemedicine adoption. This gap highlights the need for a framework integrating risks, benefits, and trust in telemedicine adoption, while addressing health care’s unique dynamics.

**Objective:**

This study aimed to adapt and extend the extended valence framework (EVF) to telemedicine, deconstructing factors driving adoption from an HCP perspective. Specifically, it investigated the nuanced roles of perceived risks, benefits, and trust referents (eg, technology, treatment, technology provider, and patient) in shaping behavioral intentions, while integrating contextual factors.

**Methods:**

We used a qualitative research design involving semistructured interviews with 14 HCPs experienced in offering video consultations. The interview data were analyzed with deductive and inductive coding based on the EVF. Two coders conducted the coding process independently, achieving an intercoder reliability of 86.14%. The qualitative content analysis aimed to uncover the nuanced perspectives of HCPs, identifying key risk and benefit dimensions and trust referents relevant to telemedicine adoption.

**Results:**

The study reveals the complex considerations HCPs have when adopting telemedicine. Perceived risks were multidimensional, including performance risks such as treatment limitations (mentioned by 7/14, 50% of the participants) and reliance on technical proficiency of patients (5/14, 36%), privacy risks related to data security (10/14, 71%), and time and financial risks associated with training (7/14, 50%) and equipment costs (4/14, 29%). Perceived benefits encompassed convenience through reduced travel time (5/14, 36%), improved care quality due to higher accessibility (8/14, 57%), and operational efficiency (7/14, 50%). Trust referents played a pivotal role; trust in technology was linked to functionality (6/14, 43%) and reliability (5/14, 36%), while trust in treatment depended on effective collaboration (9/14, 64%). Transparency emerged as a critical antecedent of trust across different referents, comprising disclosure, clarity, and accuracy. In addition, the study highlighted the importance of context-specific variables such as symptom characteristics (10/14, 71%) and prior professional experience with telemedicine (11/14, 79%).

**Conclusions:**

This study expands the EVF for telemedicine, providing a framework integrating multidimensional risks, benefits, trust, and contextual factors. It advances theory by decomposing trust referents and transparency into actionable subdimensions and emphasizing context-specific variables. Practically, the findings guide stakeholders: policy makers should prioritize transparent regulations and data security, health care organizations should provide training and support for HCPs, and technology developers must design telemedicine solutions aligning with trust and usability needs. This understanding equips health care to address barriers, optimize adoption, and leverage telemedicine’s potential for sustainable clinical integration.

## Introduction

### Background

Generally, a rise in technological innovations is omnipresent and particularly impacts the health care sector [1,2]. Nowadays, telemedicine can provide a wide range of services, which is attributed to the continuous evolution of innovations [3]. Remote patient care, diagnosis, and treatment enabled by leveraging information and communication technologies offer health care services in addition to traditional face-to-face encounters of health care professionals (HCPs) with patients [4]. The use of technology allows new opportunities to be exploited in a wide range of medical fields [5]. Among researchers, the added value, such as cost efficiency, is widely agreed upon. As an example, the percentage of global gross domestic product accounted for by health care costs is around 10% and rising [6]. Correspondingly, studies have shown that low-cost improvements in patients’ health may be achieved by using remote health services for consultation, evaluation, or treatment [7,8]. Furthermore, the implementation of telemedicine allows for increased accessibility to health care. Thus, by eliminating the proximity factor, patients can access basic and particularly specialist care from their home, which allows instant assessment or treatment [7,9-11]. The relevance of the accessibility gained via telemedicine is highlighted in diverse respects. Especially in rural areas, a shortage of health care providers represents a challenge that can be bridged by telemedicine [7]. Considering the disparity in health care among nations, the increase in telemedicine adoption has the potential to provide great benefits not only to patients but also to medical staff worldwide [10].

Despite the multitude of presented advantages, the adoption rate of telemedicine remains low [2]. Regarding diffusion, the recent COVID-19 pandemic had a strong impact on the adoption rate of telemedicine. The emergence of COVID-19 in 2020 and the restrictions that were enacted resulted in far-reaching challenges worldwide [12,13]. Telemedicine provided a viable solution to many of those challenges by avoiding direct contact, reducing the danger of transmission, and ultimately ensuring continuous care [5,14]. Consequently, the circumstances enhanced the adoption of telemedicine, inducing a rapid upscale of diffusion in 2020. However, many HCPs, especially in countries lagging in technology, remain suspicious, resulting in low telemedicine adoption rates [15]. For example, in countries such as Germany (industrialized economy), India (resource-limited economy), and Brazil (emerging economy), telemedicine has been slower to establish itself than in other countries such as Israel (industrialized economy) [16]. These countries are examples of countries lagging in technology in telemedicine adoption due to barriers such as regulatory hurdles, insufficient digital infrastructure, skepticism among HCPs, and limited access to technology in rural areas, despite the potential benefits [16,17].

Extensive research has been conducted on relevant factors for telemedicine adoption [5,18,19]. In this regard, trust has been repeatedly highlighted as a key determinant of acceptance and use of telemedicine services [20,21]. Relatedly, perceived usefulness and ease of use have also been found to influence telemedicine adoption substantially [22]. The adoption of technologies in health care brings uncertainties, particularly related to data security and privacy [23,24]. Such uncertainties often lead to perceived risks that pose significant barriers to adoption intentions [25,26]. Factors such as the HCP-patient relationship, cultural aspects, and the digital divide also serve a critical role in the overall adoption of telemedicine [27-29]. Taken together, the literature on trust, perceived risk, perceived benefits, and other factors related to telemedicine adoption has been researched in a variety of settings and from differing perspectives. Kuen et al [30], for example, highlighted the multidimensionality of risks and trust transfer effects between several trust referents in a telemedicine context from the patient’s perspective. However, it is still to be determined to what extent these constructs are also relevant from an HCP’s perspective.

The information systems (IS) literature perspective is particularly relevant for telemedicine adoption as it explores how technology is effectively implemented, managed, and accepted within organizations. Because telemedicine heavily relies on digital systems and interactions, IS literature provides valuable insights into user acceptance and integration of these technologies in health care. Indeed, several systematic literature reviews have shown that technology adoption models such as the technology acceptance model (TAM) and the unified theory of acceptance and use of technology (UTAUT) are the dominant theoretical frameworks used to study telemedicine adoption among HCPs [31,32]. While these models offer important insights into factors such as perceived usefulness and ease of use, they are limited in their ability to account for the full complexity of telemedicine adoption in health care settings. Specifically, they fail to adequately address the critical role of perceived risks, which are especially relevant due to the sensitive nature of health care services [31]. Moreover, these models often assume that the decision-making process is primarily driven by functional considerations, such as ease of integration and productivity improvements [33]. However, HCPs operate in a more complex environment, where ethical concerns, patient safety, and trust in the technology and technology provider play significant roles [30,34]. Traditional models such as TAM and UTAUT also overlook the emotional and psychological dimensions of decision-making, including anxiety about new technology or concerns about the erosion of the HCP-patient relationship [35]. In addition, these models are generally ill-suited to capture the intricate professional and legal responsibilities faced by HCPs, which can directly influence their adoption behavior [35]. In the health care context, the stakes are higher, and the consequences of technology adoption can directly impact patient outcomes, creating a level of risk that these models fail to fully incorporate [36].

Therefore, these frameworks do not offer the depth needed to explore the multilayered, context-specific factors that influence telemedicine adoption among HCPs. For example, while Bakshi and Tandon [37] explored how various dimensions of risk—such as financial, social, technological, and privacy risks—affect physicians’ intentions to adopt telemedicine, their work does not fully contextualize these risks or examine the factors that drive these perceptions. Furthermore, some studies that identify key adoption factors in telemedicine do not use specific theoretical frameworks or examine the relationships between these factors [38]. This creates a gap in the literature, as the existing models and frameworks (eg, TAM and UTAUT) do not sufficiently incorporate the multidimensional risks that are crucial in the health care context [31].

This gap underscores the need for a more comprehensive framework. While technology adoption models provide one perspective, they do not account for the broader factors that influence decision-making in health care. As Hong et al [39] argue, theoretical models must be adapted to specific contexts to provide meaningful insights. In health care, the adoption of telemedicine goes beyond technical functionality and is shaped by professional ethics, regulatory environments, and context-specific risks. Therefore, a framework that integrates these elements is needed to better explain telemedicine adoption from an HCP’s perspective. Therefore, we pose the following research question: “Which factors are relevant in the context of telemedicine adoption from an HCP’s perspective, and how can these be incorporated into an overarching framework?”

### Objectives

The research goal of this paper is to identify the factors influencing the adoption of telemedicine in terms of perceived risk, perceived benefits, and trust from an HCP’s perspective. The aim is to derive a contextualized framework for the telemedicine context, which can serve as a guideline for future research. We draw on the extended valence framework (EVF) by Kim et al [40], which was initially developed for the consumer context but has demonstrated applicability to technology adoption in various domains (eg, mobile payment solutions [41] and remote anti-doping testing [42]), including consumer acceptance of online health information services [43]. We adapt it to the context-specific characteristics of telemedicine, which include the exchange of sensitive and personal data, situations with high uncertainty and dependency for patients, the significant responsibility placed on HCPs, and the adoption of new technology.

### Theoretical Lens

**Figure 1:**
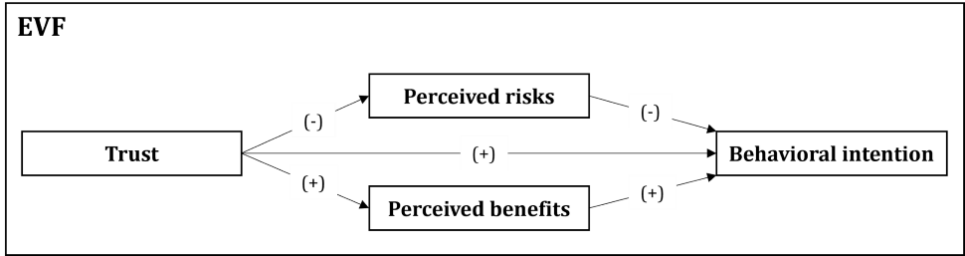
Extended valence framework (EVF) for telemedicine adoption (this depiction of the EVF is based on the study by Kim et al [40]).

To answer the research question, we draw on previous literature on adoption behavior and provide a brief introduction to the relevant definitions of the related constructs. We argue that the EVF is well suited as an overarching framework to explore relevant factors for technology adoption as it provides a broad perspective on individuals’ attitudes and perceptions toward a new technology [40]. Unlike traditional models such as the TAM and the UTAUT, which primarily emphasize positive factors such as perceived usefulness, the EVF integrates both positive drivers (eg, perceived benefits) and negative drivers (eg, perceived risks), offering a more balanced understanding of adoption decisions. This is particularly critical in health care, where concerns over patient safety, data security, and ethical implications play a central role in decision-making. The EVF integrates perceived risks, perceived benefits, trust, and behavioral intentions, all of which are critical to the adoption of new technologies [44]. Figure 1 [40] depicts the layout of the EVF in which trust positively affects perceived benefits and behavioral intentions but negatively affects perceived risks. In addition, perceived risks have a negative relationship, and perceived benefits have a positive relationship with behavioral intentions.

In the context of telemedicine, the EVF is particularly appropriate because it not only captures the benefits of new technology but also addresses the risks associated with it, such as misdiagnoses, technical reliability, and patient confidentiality, which are often underexplored in models such as TAM and UTAUT. Perceived risk refers to the uncertainty and potential severity of negative consequences, a definition established by Dowling and Staelin [45] as well as Featherman and Pavlou [26]. Various dimensions of risk have been identified in the literature, underscoring its multifaceted nature [46]. While the risk dimensions used in this study were initially developed in consumer e-services and purchasing contexts [26,47,48], research has extended their applicability to health care and telemedicine [30]. Table 1 provides an overview of the risks identified by previous research and their respective definitions.

**Table 1:** Definitions of risk dimensions relevant to telemedicine adoption.

Risk dimension	Definition	Study
Performance risk	Reflects the perception that a product or service fails to meet the anticipated performance standards, consequently not delivering the desired benefits	Grewal et al [47], 1994
Privacy risk	Reflects the “potential loss of control over personal information”	Featherman and Pavlou [26], 2003
Financial risk	Reflects the potential monetary outlay due to the product’s or service’s costs	Featherman and Pavlou [26], 2003
Psychological risk	Reflects the mental stress that possibly arises through the use of technology	Jacoby and Kaplan [48], 1972, and Featherman and Pavlou [26], 2003
Time risk	Reflects the potential loss of time when making a bad purchasing decision by wasting time researching and making the purchase, learning how to use a technology or service only to must replace it if it does not perform to expectations	Featherman and Pavlou [26], 2003
Social risk	Reflects the potential loss of status in one’s social group because of adopting a product or service, looking foolish or untrendy	Featherman and Pavlou [26], 2003

Contrary to the multidimensional definition of perceived risk, perceived benefit represents the extent to which an individual believes that they will benefit from using a product or service [23]. It is characterized by the user’s motivation to maximize the expected value associated with a technology [40]. Therefore, in this study, perceived benefit is defined as the potential value that a user associates with the use of telemedicine. As telemedicine represents a context that is different from other topics addressed in IS research, commonly used constructs cannot be easily transferred [49]. Previous research on telemedicine has shown, for example, the impact of efficiency on perceived benefits in health contexts such as autism and diabetes [7,8], as well as the relevance of the perceived quality of the specific service or product under consideration [50-52]. A study on the patient’s perspective toward telemedicine adoption additionally highlights convenience as an impacting factor [53].

Within the EVF, trust is integrated as another relevant variable, as studies have shown that it reduces risk perceptions and can increase perceived benefits [54-56]. Thus, we include it in the scope of our contextual decomposition approach and define trust according to Mayer et al [57] as “the willingness of a party to be vulnerable to the actions of another party based on the expectation that the other will perform a particular action important to the truster, irrespective of the ability to monitor or control that other party.” Within this definition, a relationship is considered to exist with another identifiable party that acts and reacts with volition toward the truster. Both parties—the truster and the trustee—are not limited to individuals; they can also include other entities, both physical and non-physical, such as technology [58]. Interpersonal trust is considered to be a trusting relationship between individuals. According to Mayer et al [57], individuals’ trust is determined by their perception of the trustworthiness of the other party. Trustworthiness is considered a multidimensional construct, the dimensions (ability, benevolence, and integrity) that influence trust in another individual, groups of individuals, or an organization and lead to a higher willingness to take risks in the respective relationship [57]. In addition to interpersonal trust, the need to distinguish trust in technology emerged. While trust in an individual entails a moral and conscious interpersonal dynamic, technology is an artificial creation with limited capabilities and no moral agency. Therefore, further research on trust developed a multidimensional construct of trusting beliefs in technology [55,59]. Table 2 shows a compromised overview of the relevant dimension definitions.

As this study aims to decompose the EVF, we look at several potential and context-specific trust referents. In research on trust in telemedicine, 4 main stakeholders have been identified (technology, treatment, patient, and provider) [60]. The concept of trustworthiness and trusting beliefs can be applied to patients and providers (trustworthiness) as well as technology (trusting beliefs). As treatment is understood to be a process, it does not fall under the 2 trust constructs. However, trust in treatment can be characterized as an individual’s belief that the telemedical treatment effectively addresses medical concerns, is clear, and results from a collaborative decision-making process between the patient and HCP [30,61].

**Table 2:** Definitions of trust dimensions for interpersonal and technological contexts.

Trust construct and dimension			Definition		Study	
**Interpersonal trust/trustworthiness**						Mayer et al [57], 1995
	Ability	The skills, competencies, and attributes that enable a party to have an impact in a specific domain.				
	Benevolence	The degree to which a trustee intends goodwill toward the truster, alongside their perceived positive attitude toward the truster.				
	Integrity	The truster’s perception that the trustee adheres to a set of principles that the truster finds acceptable.				
**Trust in technology/trusting beliefs**						McKnight et al [59], 2011
	Functionality	The belief that a specific technology has the appropriate functionalities or features to fulfill the requirement.				
	Reliability	The belief that the specific technology will consistently operate properly.				
	Helpfulness	The belief that adequate and responsive help is provided by the technology.				

## Methods

### Study Design

To determine how perceived risks, perceived benefits, and trust are related in the specific context of telemedicine, we referred to the guidelines by Hong et al [39] on context-specific theorizing. Accordingly, we chose a qualitative research approach and conducted semistructured interviews with HCPs who already offer video consultations to their patients, which is considered a widely applied method to obtain contextual and authentic accounts [62]. HCPs represent the primary party in adopting telemedicine, as they must be willing to offer telemedicine to enable patients to actively opt for it. Hence, they have the opportunity to shape the broad adoption of telemedicine [63]. Given the limited adoption of this technology, interviewing HCPs with extensive experience in telemedicine is expected to provide greater value, as their use and familiarity with it are essential for overall social acceptance.

As part of the study’s rigorous design, a pilot study was conducted before the actual data collection to ensure the clarity and relevance of the interview guide [64]. This pilot phase involved conducting preliminary interviews with 4 HCPs who had prior experience with telemedicine. On the basis of this feedback, the interview guide only had to be marginally adapted. The interview guide was mainly guided by the EVF (Figure 1), covering key constructs such as trust, perceived risks, perceived benefits, use behavior, and intention (Table S1 in Multimedia Appendix 1). It also included contextual factors to capture additional insights that may not be covered by the EVF. The guide consisted of open-ended questions designed to explore how these factors influence telemedicine adoption from the HCP’s perspective. This structure allowed for flexibility, enabling follow-up questions to be asked during the interviews as needed to explore emerging themes.

On the basis of the EVF, a combination of deductively derived and inductively elaborated relevant constructs was chosen as the research approach [65]. Thus, in line with the study by Hong et al [39], the various constructs can be decomposed, the appropriateness of the EVF for the specific context can be examined, and all relevant constructs can then be transferred into a context-specific variation of the EVF. In addition, newly emerging antecedents and context-specific variables are added.

### Data Collection

To ensure the interview partners were sufficiently qualified, only HCPs who (1) have completed formal education in the medical or therapeutic field, (2) have experience with telemedicine through video consultations, and (3) practice in Germany (to ensure cultural comparability) were contacted. All medical specializations were included. As psychotherapists represent about 25% of HCPs in Germany, this group was included in our analysis [66]. This selection deliberately focused on gaining insights from HCPs who have practical experience with telemedicine, enabling a deeper exploration of its real-world effectiveness and challenges. Involving only experienced users ensures that the study captures practical insights that are directly relevant to improving the future implementation of telemedicine. Obtaining consent from interviewees and assuring anonymization, we audio-recorded the interviews and then transcribed them verbatim [67,68]. We chose to recruit via the medical website Jameda [69]. A total of 142 HCPs who met the requirements as experts were contacted by email. Out of these 142 professionals, 26 (18.3%) expressed interest in participating. The interviews were conducted online via Zoom (Zoom Video Communications) during March and April 2023. After 14 interviews, theoretical saturation was reached, suggesting that further interviews would be unlikely to add any new insights [70].

### Sample Description

The resulting interview sample included 14 experts, half of whom were male (7/14, 50%) or female (7/14, 50%) and were, on average, aged 47 (range 34-68) years. The length of the interviews varied between 13 and 53 (mean 27, SD 10) minutes. Table 3 gives an overview of the relevant descriptives.

**Table 3:** Characteristics of the 14 health care professionals interviewed.

Number	Age (y)	Highest education	Field of practice
1	43	Doctoral degree	Palliative care
2	49	High school diploma	Psychotherapy
3	55	High school diploma	Psychotherapy
4	48	Doctoral degree	Gynecology
5	68	Master’s degree	Psychotherapy
6	45	Doctoral degree	Psychiatry and psychotherapy
7	52	Doctoral degree	General medicine
8	50	Doctoral degree	Endocrinology
9	36	Bachelor’s degree	Physiotherapy
10	42	Doctoral degree	Gynecology
11	68	Doctoral degree	General medicine
12	37	State examination	Psychotherapy
13	35	Doctoral degree	General medicine
14	34	State examination	General medicine

### Data Analysis

We conducted a qualitative content analysis [65] using MAXQDA 2022 (version 22.7.0) software. The analysis was conducted by 2 authors (coder A and coder B) separately and using deductive as well as inductive coding methods. The deductive codes were derived from the relevant constructs already outlined in the theoretical lens based on the EVF. First, all transcripts were coded by coder A according to these categories. In addition, coder A inductively derived subcategories to allow for more in-depth explanations and context-specific extension, which were then discussed with coder B. Following coding, coding rules, definitions, and anchor examples were developed to ensure consistency for each category. Coder B performed the coding process independently following coder A. Subsequently, the coding was iteratively extended and analyzed in several steps. Transparency, for example, was identified as an antecedent to trust and coded following the dimensions defined by Schnackenberg and Tomlinson [71] (disclosure, clarity, and accuracy). We adhered to the standards set by Venkatesh et al [72] to ensure the quality of our qualitative research. The validity of our research design was confirmed through a thorough account of our research context, which increased the relevance and trustworthiness of our conclusions. To ensure a valid coding process, we took steps to ensure inferential validity. The interauthor agreement in the coding process resulted in an intercoder reliability of 86.14%. To account for the potential of random agreement at the segment level, we assessed coding reliability and calculated Cohen κ, which yielded a value of 0.86. These results suggest an “almost perfect” agreement between the 2 coders [73].

### Ethical Considerations

The study received ethics approval from the ethics committee of the School of Business and Economics at the University of Münster (2020–02). The research adhered to ethical guidelines for human subject research. All participants provided written informed consent before participating in the study. The consent process included an explanation of the study objectives, procedures, and the right to withdraw at any time without any consequences. To protect participants’ privacy, all interview data were anonymized during transcription. Identifiable information was removed, and participants were assigned unique codes to ensure confidentiality. Data were securely stored on encrypted devices accessible only to the research team. No monetary or material compensation was provided to participants for their involvement in the study. This study does not include any images of participants or supplementary material that could potentially identify individuals. As a precaution, the interview transcripts were carefully reviewed to exclude any inadvertent references that could lead to participant identification.

## Results

### Outcome Evaluation

By decomposing the constructs of the EVF, our results demonstrated their individual sublevels and relevance to the context of telemedicine. We found that HCPs clearly perceived both the risks and benefits of telemedicine. They were able to reflect on and consider these extensively. In addition to the decomposed constructs of the EVF, we identified context-specific variables; transparency was an inductively derived factor that preceded trust. The following sections presents our result according to each construct of the EVF. Although certain inductive codes were mentioned infrequently, their inclusion is justified by the nature of qualitative research. Qualitative analysis values the depth of insights over frequency, meaning even low-frequency mentions can offer significant contextual relevance. These rare codes can highlight emerging or underexplored challenges within telemedicine adoption, particularly in niche areas of health care practice [74]. Specific quote examples for the inductively generated level 3 codings can be found in Tables S2-S6 in Multimedia Appendix 1.

### Perceived Risks

Limited treatment options due to visual restrictions were mentioned with regard to *performance risk*; that is, no holistic insight into the patient can be gained. Furthermore, physical contact is omitted, and further examinations for diagnosis might be neglected. This results in the “...danger...that something is overlooked in the diagnosis” (interviewee 11). Internet connection issues were additionally noted, especially concerning patients in rural areas. An interviewee expressed the following:

...the great issue is the poor internet connection of the clients most of the time....

Interviewee 4

In addition, *financial risks* were recognized, particularly associated with acquiring high-technology equipment. While the general cost of equipment was not seen as a risk, the purchase of advanced, high-technology tools was perceived differently. An interviewee stated the following:

You could also control it remotely and make a much better zoom than with the webcam. Such a tool is expensive, of course.

Interviewee 6

Concerning *time risks*, the investment of time required for staff to familiarize themselves with technology and its functions was underlined. One respondent mentioned that “[Telemedicine] is indeed a considerable investment of time, as you have to train the staff as a whole” (interviewee 10), pointing out the significant time commitment necessary for staff training. As far as *psychological risk* is concerned, it was rarely addressed and, if so, was associated with an uncomfortable feeling and a certain weight in digital communication. Concerning *social risks*, it was inferred that negative comments are mainly received from the social and professional environment. However, *privacy risk* was most frequently addressed by the interviewees. The main focus was on the perceived risk posed by patient data security:

The basic question is always that of data security since patient data is exchanged.

Interviewee 1

Table 4 shows the deductive and inductive codes for perceived risks according to the number of interviews in which these were addressed.

**Table 4:** Results of the qualitative content analysis for perceived risks (N=14).

Levels 1^a^, 2^b^, and 3^c^				Interviews in which this aspect was mentioned, n (%)
**Perceived risk**				
	**Performance risk**			
		*Restriction in treatment* ^d^	7 (50)	
		*Restriction in patient-HCP* ^e^ *connection*	7 (50)	
		*Internet connection issues*	5 (36)	
		*Technical issues*	4 (29)	
		*Technical proficiency of patient*	5 (36)	
	**Financial risk**			
		*Need for equipment*	4 (29)	
		*Need for staff*	1 (7)	
		*Insurance coverage*	3 (21)	
		*Limited user group*	2 (14)	
		*Inadequate compensation*	1 (7)	
	**Time risk**			
		*Familiarization with the technology*	7 (50)	
		*Adaptation to the new workflow*	2 (14)	
		*Research for alternatives*	1 (7)	
	**Psychological risk**			
		*Uncomfortable feeling*	3 (21)	
		*Weight of communication*	3 (21)	
		*External interfering factors*	2 (14)	
	**Social risk**			
		*Negative comments*	5 (36)	
	**Privacy risk**			
		*Data security*	10 (71)	
		*HCPs’ privacy*	2 (14)	
		*Patients’ privacy*	4 (29)	

^a^Category derived from the study by Kim et al [40].

^b^Categories derived from the study by Featherman and Pavlou [26].

^c^Categories are inductive codes based on specifications from respondents.

^d^Inductive codes are italicized.

^e^HCP: health care professional.

### Perceived Benefits

Interviewees expressed multiple benefits related to the *convenience of telemedicine*. A notable advantage was the reduction in travel time, which benefited HCPs by allowing them to manage their schedules more efficiently and handle a higher volume of consultations. One participant stated that video consultation had the following benefits: “No more traveling. Rehabilitation clinics, in particular, are always a little further out of town as far as our field is concerned*.*” (interviewee 9). Moreover, convenience was accentuated in treating patients located in rural areas. Another interviewee emphasized that these patients particularly value telemedicine as it allows them to receive consultations at home. In addition, interviewees commented on the medical implications of reduced travel, mentioning that longer travel durations “may also not be harmless due to the illness” (interviewee 6). They suggested that maintaining distance, especially if patients are contagious, is sensible for both patient and HCP. Apart from convenience, there was a shared sentiment that telemedicine augments the *quality of care*. Accessibility was often spotlighted as a distinct advantage of telemedicine, with interviewees noting its significant role in improving patient care. In addition to the accessibility of medical treatment and consultation, the possibility of maintaining a safe space was mentioned several times, particularly in the psychological field, as many patients might feel more comfortable at home:

...the more distance there is between medical staff and patients,...it gives patients a low-barrier opportunity to mention things that they might not dare to mention face-to-face.

Interviewee 9

Consequently, HCPs obtained more relevant information, thus ensuring a higher quality of diagnosis and treatment. Furthermore, telemedicine was observed to enhance HCP’s *efficiency*. The emphasis was largely on the significant time savings for HCPs, allowing them to provide timely care while easily coordinating and managing their schedules through health platforms. This streamlined process enables HCPs to handle more appointments and optimize their workflow, ultimately improving their productivity. Table 5 shows the deductive and inductive codes for perceived benefits according to the number of interviews in which these were addressed.

**Table 5:** Results of the qualitative content analysis for perceived benefits (N=14).

Levels 1^a^, 2^b^, and 3^c^				Interviews in which this aspect was mentioned, n (%)
**Perceived benefit**				
	***Convenience of telemedical use ^d^***			
		*Space independence*	8 (57)	
		*Reduction of travel time*	5 (36)	
		*Time flexibility*	6 (43)	
		*Protection of HCPs’* ^e^ *well-being*	4 (29)	
		*Protection of patients’ well-being*	6 (43)	
	***Quality of care***			
		*Accessibility*	8 (57)	
		*Safe space for sensitive topics*	4 (29)	
		*Enhanced perception*	3 (21)	
	***Efficiency of telemedical service***			
		*Time saving*	7 (50)	
		*More precise working time*	2 (14)	
		*Financial advantage*	4 (29)	

^a^Category derived from the study by Kim et al [40].

^b^Categories derived from the study by Featherman and Pavlou [26].

^c^Categories are inductive codes based on specifications from respondents.

^d^Inductive codes are italicized.

^e^HCP: health care professional.

### Trust Referents

Trust referents were distinguished by the HCPs, and all were acknowledged, although not all were considered to be of high importance. Trust in technology was typically mentioned in terms of its functionality, such as the ability to provide audio and visual connection, schedule appointments, and even collect some anamnesis data; for example,

[Take] a quick picture of the throat with a smartphone so that you can get an idea. Are the tonsils swollen now? Are they coated? That works.

Interviewee 13

With regard to *trust in the treatment process*, 3 subcategories emerged, which can be subdivided into the initial necessary consensus, the subsequent effective collaboration of patient and HCP, and the final effectiveness of the treatment. The patient-HCP connection was particularly relevant for HCPs:

Definitely, if you manage to maintain the personal factor further [using video consultation].

Interviewee 9

Trust in the provider was supported above all by the data security it offers, which also corresponds to the high perceived risk of data security:

I trust my specialist provider, so to speak, and assume that the solution he offers has also been checked with the data protection authorities in terms of data protection law.

Interviewee 2

It also became clear that the provider is expected to have both technical competence and knowledge of medical procedures, that is, an understanding of the particular application context. Compared to the other trust referents, the HCPs’ perception of *trust in the patient* was rather limited. It was primarily based on the digital affinity assigned to the patient, which is relevant for the HCP to be able to use telemedicine effectively together with the patient. Table 6 presents the deductive and inductive codes for all trust referents.

**Table 6:** Results of the qualitative content analysis for trust referents (N=14).

Levels 1^a^, 2^b^, and 3^c^				Interviews in which this aspect was mentioned, n (%)
**Technology as a trust referent**				
	**Technology’s functionality**			
		*Audio connection* ^d^	5 (36)	
		*Visual connection*	6 (43)	
		*Appointment scheduling*	1 (7)	
		*Anamnesis data taken*	3 (21)	
	**Technology’s reliability**			
		*Stable connection*	5 (36)	
		*Stable platform*	4 (29)	
**Treatment as a trust referent**				
	***Consensus understanding of the treatment process***			
		*Practicality*	4 (29)	
		*Enabling adequate dialogue*	4 (29)	
	***Effectiveness of collaboration***			
		*Consent of patient*	5 (36)	
		*Enabling compliance*	4 (29)	
		*HCP ^e^-patient connection*	9 (64)	
		*Patient feedback*	2 (14)	
	***Effectiveness of treatment***			
		*Provision of medical reports*	3 (21)	
		*Work facilitation*	4 (29)	
		*Possible guidance to self-examination*	2 (14)	
**Provider as a trust referent**				
	**Provider ability**			
		*Contextual knowledge*	2 (14)	
		*Technical knowledge*	2 (14)	
	**Provider benevolence**			
		*Proximity*	1 (7)	
	**Provider integrity**			
		*Data security*	11 (79)	
		*Responsiveness*	3 (21)	
		*Certification*	3 (21)	
**Patient as a trust referent**				
	**Patient ability**			
		*Digital affinity*	5 (36)	

^a^Category derived from the studies by Van Velsen et al [60] and Kim et al [40].

^b^Categories derived from the studies by Mayer et al [57] and McKnight et al [59].

^c^Categories as inductive codes based on respondents’ specifications.

^d^Inductive codes are italicized.

^e^HCP: health care professional.

### Transparency

In the coding process, transparency emerged as an important antecedent to several trust referents for HCPs. This is in line with previous literature that identified transparency as an antecedent to trust, as information shared in this way indicates the ability, benevolence, and integrity of a truster [75,76]. The definition by Schnackenberg and Tomlinson [71] is widely accepted and defines transparency as “the perceived quality of intentionally shared information from a sender.” We followed the accepted multidimensional approach and accordingly subdivided transparency into its 3 dimensions: disclosure, clarity, and accuracy [71,76,77]. Thereby, we define “disclosure” as the belief that all relevant information is shared in a timely manner, “clarity” as the level of correspondence between the intended and the understood meaning of information, and “accuracy” as the extent to which information given matches reality and is free from intentional distortions [71,76].

Transparency was emphasized in the information provided about technology, treatment, and providers. The patient as a trust referent was not mentioned when it came to transparency. In addition to transparency in relation to the provider, the relevance for disclosure on technology also became apparent: “Now I’m not a technician and can’t assess what it could do with this data, but I feel well informed.” (interviewee 3) Similarly, the importance of transparency in the treatment process between the patient and the HCP was highlighted: “When you feel like you’ve had a lot explained to you and you have a lot of knowledge about it and you know how things work” (interviewee 9). Table 7 shows the deductive codes for the transparency recipients according to the number of interviews in which these were addressed for each transparency dimension.

**Table 7:** Results of the qualitative content analysis for transparency dimensions (N=14).

Levels 1, 2^a^, and3^b^				Interviews in which this aspect was mentioned, n (%)
**Transparency**				
	**Transparency on technology**			
		Disclosure	2 (14)	
		Clarity	1 (7)	
		Accuracy	3 (21)	
	**Transparency on treatment**			
		Disclosure	4 (29)	
		Clarity	2 (14)	
		Accuracy	3 (21)	
	**Transparency on provider**			
		Disclosure	7 (50)	
		Clarity	7 (50)	
		Accuracy	5 (36)	

^a^Categories are in accordance to the trust referents based on the studies by Van Velsen et al [60] and Kuen et al [30].

^b^Categories derived from the study by Schnackenberg and Tomlinson [71].

### Context-Specific Variables

Regarding the need for contextual specification mentioned by Hong et al [39], we were able to identify variables that apply specifically to the telemedicine context. Especially, the relevance of prior consideration of the symptom characteristics and their suitability for treatment with telemedicine services, such as video consultation, was highlighted:

I can take a lot of responsibility for doing this via telemedicine or not....And I have a bad feeling when I hear patients report that...the patient is being sealed off even more.

Interviewee 12

It is not so much the distinction between mental and physical that plays a role here, but rather the necessary examination proximity and physical treatment approach:

What is more difficult is to examine you....Let’s say one would have to palpate it or something else, then it stops.

Interviewee 1

Another context-specific variable is the previous experience or familiarization of the HCP and their patients with telemedicine:

[Video consultation] has also now worked out quite well over the last two years.

Interviewee 8

Frequent positive experiences, therefore, increase both the willingness to adopt and the level of trust in the technology. Table 8 shows the inductively derived codes for context-specific variables.

**Table 8:** Results of the qualitative content analysis for context-specific variables affecting telemedicine adoption.

Levels 1 and 2^a^		Interviews in which this aspect was mentioned, n (%)
**Context variable**		
	Symptom characteristics (10)	10 (71)
	Experience (11)	11 (79)

^a^Categories derived inductively.

**Figure 2:**
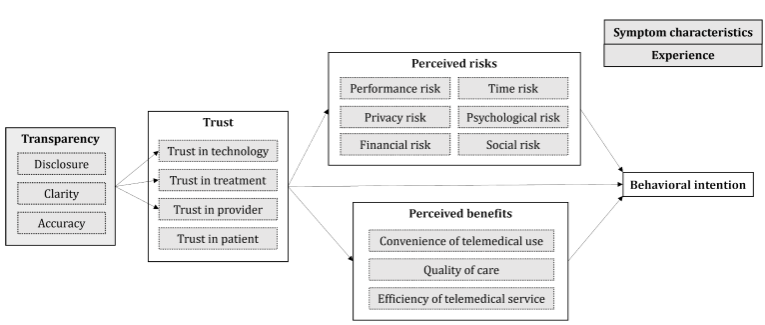
Telemedicine-contextualized valence framework. The extended valence framework is based on the study by Kim et al [40]. Perceived risk categories follow the study by Featherman and Pavlou [26]. Trust referents are derived from the studies by Van Velsen et al [61] and Kuen et al [30], and transparency dimensions are based on the study by Schnackenberg and Tomlinson [71].

In summary, our proposed expansion of the EVF is shown in Figure 2 [26,30,40,60,71]. We consider transparency a key multidimensional antecedent, along with context-specific variables such as symptom characteristics and experience with telemedicine services.

## Discussion

### Interpretation of Results

Our study provides a multifaceted examination of the considerations HCPs have when adopting and using telemedicine services, revealing various facets of perceived risks and benefits, trust referents, transparency, and context-specific variables.

First, it is evident that HCPs have various concerns about telemedicine. Concerns primarily relate to performance risks such as visibility limitations that restrict holistic insight into the patient’s well-being. Lack of physical contact and potential confusion in diagnosis were highlighted by the HCPs, pointing to the critical role of traditional face-to-face interaction in medical assessments. Financial risks, such as the time required to learn to use technology, and privacy risks, such as concerns about data security, emphasize the challenges that HCPs face. Previous studies have identified technological issues, data security, and privacy as primary risks associated with the telemedicine context [30,78]. Consistent with earlier research, this study reaffirms the existence and multidimensionality of perceived risk [26,30], further delineating it into subdimensions of performance risks (eg, visual restrictions), privacy risks (eg, data security), and financial risks (eg, uncertainty about insurance coverage) as the risks most often mentioned. The addition of these context-specific subdimensions is a nuanced insight, adding depth to the understanding of practical challenges faced by HCPs.

Second, among the perceived benefits, the convenience of the telemedical approach, quality of care, and efficiency of the telemedical service were identified as key factors. Particular emphasis was placed on minimizing the HCP’s travel time and improving the protection of the patient’s well-being by reducing the need for transportation. This convenience, along with increased comfort and protection through minimal physical contact, establishes a better experience and better safety using telemedicine. In addition, our findings indicate that telemedicine can foster an environment that leads to increased efficiency of the services the HCP offers. Telemedicine was often complimented for its convenience, accessibility, and potential to deliver health care services to remote or underserved areas [7]. Increased patient engagement and satisfaction were also commonly cited benefits [10]. Our results align with existing literature, emphasizing convenience, quality, and efficiency as significant benefits and adding context-specific dimensions by highlighting the potential for streamlined operations and enhanced HCP efficiency in subdimensions.

Third, we found that trust is a key driver of adoption behavior, with multiple trust referents such as technology, treatment process, provider, and patient. Our findings illustrate that while technology and treatment process were strongly and diversely perceived trust referents, trust in patients was solely measured by the patient’s abilities in terms of their digital affinity. This underscores the important role that technical reliability and effective treatment collaboration have in successful telemedicine practices. These findings corroborate prior research, signifying trust as a multifaceted concept involving technology, treatment processes, and patients [30,60]. However, our results contradict the relevance of trust in patients from an HCP’s perspective, as they represent a given parameter in the general treatment process for the HCP, and changing to an online setting seems to adapt the relationship only to the extent that patients must be able to use the new technology in question. Furthermore, the subdivisions of the treatment process need to be considered. Therefore, relevancy for trust is seen in particular in the second part, the consensus of collaboration. This is consistent with the assumption that the highest level of interaction and reliance on functioning communication between patient and HCP occurs here. Therefore, this study offers a more nuanced perspective by identifying the different relevancy of trust referents from the HCP’s perspective.

Fourth, transparency emerged as an antecedent for trust, indicating the need for disclosure, clarity, and accuracy in technological processes and interactions with providers to foster a trustworthy telemedicine environment. This underscores the critical role of clear communication and information sharing. Transparency, specifically regarding data use and security, has been identified as crucial in previous research in differing contexts [79-81]. In addition, transparency is often linked to increased trust and acceptance of telemedicine services [60]. Notably, the need for transparency was identified for all trust referents except the patient. This could be explained by the personal view of the HCPs, in that they do not feel any dependency on their patients, as they perceive themselves as service providers on whom the patients depend. This highlights the special nature of the medical sector and the attitude and unilateral dependent relationship between HCPs and patients. Our results align with prior research, highlighting the importance of transparency as an antecedent for certain trust referents. This underscores the need to tailor the information provided according to the HCPs’ relevant trust referents when fostering a trustworthy telemedicine environment. We also show that the view of transparency as multidimensional and the 3 defined dimensions (disclosure, clarity, and accuracy) [71] are transferable to the context under consideration and that, with a qualitative research approach, no major deviations in the relevancy of the individual dimensions are apparent.

Fifth, context-specific variables such as symptom characteristics and prior experience with telemedicine have a significant role in its use and efficacy. An HCP’s intention to use telemedicine is influenced by various contextual factors such as the characteristics of symptoms, patient demographics, and technological familiarity among users [82-84]. The results from our interview study resonated with these findings, emphasizing the impact of symptom characteristics and previous telemedicine experiences on its use and perceived effectiveness. In addition, adoption behaviors may vary across medical specializations, depending on how telemedicine software interacts with clinical workflows and the patient-practitioner dynamic. For example, therapy with clinical psychologists might involve a more personal and sensitive experience compared to discussions of physical symptoms with HCPs, which may not require the same level of intimacy. The distinct benefits telemedicine can offer in fields such as infectious diseases further highlight the need to consider how different clinical contexts impact telemedicine adoption. Thereby, we specify which type of symptom characteristic is decisive for the usability of telemedical services such as video consultation. We abandon a division into mental and physical symptoms, an approach taken in prior research [30], as we found no significant difference between mental and physical symptoms. Instead, distinctions should be made according to the severity of the need for direct intervention. This is similar to a distinction of symptom severity, which has also already been introduced [85], but should not be interpreted as synonymous with it. The demonstrated impact of repetition, leading to increased experience and familiarity, is consistent with other studies, such as the study by Venkatesh et al [33], which highlight the importance of the repetition cycle in building trust [86].

### Implication for Research

Several theoretical implications can be derived from the results of our study. Arguably, the first and most straightforward is the applicability and relevance of the guidelines by Hong et al [39] for context-specific theorizing. Our results show that a systematic decomposition of the relevant constructs of adoption behavior should address context-specific characteristics, especially because the context of telemedicine is characterized by a special relationship among the patient, HCP, and technology used for the treatment process and its provider.

Our second theoretical contribution is that this study addresses the limitations of existing technology adoption models such as TAM and UTAUT by incorporating telemedicine-specific factors into a contextualized framework. While TAM and UTAUT focus primarily on general constructs such as perceived usefulness and ease of use, they often fail to capture critical dimensions relevant to health care, such as the multifaceted risks and trust referents that are crucial for HCPs. The developed contextualized model overcomes these limitations by integrating additional factors such as transparency and symptom characteristics, offering a more comprehensive understanding of telemedicine adoption from an HCP perspective. This framework provides a valuable additional perspective to existing technology adoption models, serving as a foundation for future research on telemedicine adoption. Moving forward, future research can build on this by conducting quantitative validation and refining the framework further.

Third, our study emphasizes the multidimensionality of relevant factors in telemedicine, such as risks [26,30] and trust [59,60]. Recognizing the multidimensional nature of these factors enables a more nuanced understanding and specific contextualization for telemedicine. This perspective can guide future research to explore these dimensions in different health care settings and with different stakeholders.

Fourth, the developed framework offers starting points for further analysis of stakeholders in health care. For example, the perspectives of patients or technology providers, who have already been identified by Van Velsen et al [60] as trust referents in the context of telemedicine adoption, may be examined in future studies. Understanding their perspectives and relevant context-specific factors can enrich the theoretical understanding of telemedicine adoption and its broader implications in health care.

### Practical Implications

Several practical implications for HCPs and their use of telemedicine can be derived from our findings. First, the need for additional training of medical staff in telemedicine technologies can be addressed by ensuring that HCPs can focus on medical examinations and treatments without being distracted by technical issues. Therefore, health care facilities and general practices should consider hiring specialized telemedicine support staff. This approach enables a more efficient division of labor and ensures that technical problems do not affect the quality of medical care. In addition, specific training programs can be developed or used to help staff mitigate risks and fully understand and leverage the benefits of telemedicine. Training should thus include best practice approaches and encourage exchange with other medical facilities. In addition, telemedicine should be formally integrated into medical school curriculums and clinical placements to prepare future HCPs for the evolving landscape of digital health care. This will ensure that medical students are equipped with both the technical and clinical skills necessary for using telemedicine effectively in their practice. However, it is important to note that the cost and feasibility of these recommendations may vary, particularly for smaller or resource-constrained organizations. Ideally, telemedicine software should be intuitive enough for existing staff, such as IT or administrative personnel, to manage with minimal training, although this may not always be feasible.

Second, the individually identified risks that are particularly relevant can be addressed in a more targeted manner. To better manage such risks, risk management guidelines could be developed to provide guidance and protocols to counteract the perceived risks of telemedicine, for example, by reducing the performance risk through enhanced remote examination with improved imaging technologies or remote monitoring tools to mitigate visibility limitations and diagnostic confusion. Data security protocols can also be strengthened, thus minimizing the privacy risk, as insurance with compliance privacy regulations sends a trustworthy signal.

Third, transparency is a significant antecedent that underlines the necessity to choose an appropriate provider and the right technology for the planned medical approach. On the basis of our results, HCPs should choose telemedicine platforms and technologies that fit their most important trust factors, for example, those that offer reliable, user-friendly interfaces and robust security features. Thus, professionals should look for providers that offer comprehensive support and training. In addition, health care institutions should aid HCPs in selecting the appropriate telemedicine platform and technology by choosing their recommendations based on the criteria that emerged as particularly relevant in this study.

Fourth, as not every symptom characteristic can be treated equally well or at all with telemedicine, providers should focus on developing solutions that are specialized for certain medical conditions and symptoms and can optimally support these. By tailoring their technologies and programs to diverse symptom characteristics, HCPs may more effectively manage treatment via telemedicine. However, developing specific policies and guidelines on when it is appropriate to use telemedicine may be a more practical approach, given the constraints of resources and the limitations of digital communication. These guidelines can help ensure that telemedicine is used optimally without compromising patient care.

### Limitations and Further Research

First, the amendments to the framework are based on a qualitative approach that aimed to identify relevant constructs for the considered context. Next, they should be tested quantitatively for the strength of the proposed causal relationships and applied to other telemedicine applications, such as telemonitoring or videoconferencing between HCPs that are not diagnostic or a treatment. We have added the new context-adapted subcategories to already established constructs, such as multiple risks. Therefore, future research should review and adapt existing scales to ensure that they cover the relevant new subcategories. However, the current framework does not fully address key human factors such as task load burden and the user experience of interacting with telemedicine software in daily clinical practice. These factors represent ongoing, structural impacts on health care delivery rather than the transitional costs of implementation. Although this was not a focus of this study, it is important to acknowledge this limitation and consider it as an avenue for future research.

Second, we adjusted the sample size to suit the rarity of the niche population (ie, HCPs in Germany offering video consultations). Due to the low adoption rate, HCPs with an affinity for technology are more likely to use telemedicine. Therefore, the benefits could be overestimated and the perceived risks underestimated. In addition, the use of volunteer sampling may introduce self-selection bias, as those who choose to participate are likely to hold more favorable and supportive views of telemedicine. This could further increase the risk of overestimating its benefits and underestimating its risks. Thus, it has to be argued that there is a possible lack of generalizability of our results, which should be addressed in future studies by considering larger samples and subject triangulation [87]. However, we intentionally only interviewed HCPs with experience, ensuring that the perceived benefits and risks were based on real experience rather than assumptions. Moreover, we ensured a diverse sample in terms of age, gender, and specialization to take a variety of perspectives into account. Future research should compare our findings with the perspectives of HCPs who have not adopted telemedicine as well as their transferability to a patient’s perspective. Furthermore, the transferability of our results to the patient’s perspective ought to be examined, given that previous studies showed a greater disparity between HCPs and patients [88]. This will provide deeper insights into the barriers and facilitators to further implementation of telemedicine.

Third, our sample is limited to the German context with its national regulations and funding opportunities. Cultural factors specific to Germany, such as high individualism and low uncertainty avoidance [89], may also influence perceptions of telemedicine. These biases could differ significantly in other cultural environments, particularly in low-income countries, where telemedicine adoption might be shaped by different cultural attitudes and resource constraints. Telemedicine is already more established in other countries, which might differ in terms of regulatory and organizational conditions [90,91]. Our study shows the specificity of early adopters, who may still face more prejudices, insecurities, and lack of habits. The results of this study can be adapted to other cultural environments with more established telemedicine offerings and reveal differing cultural-specific results. To address the relevance of the governmental framework, future research should analyze how recent and anticipated changes in health policy and regulations affect the adoption and effectiveness of telemedicine and how this might affect the constructs proposed in this study’s adoption framework. In addition, the demographics and communities served by telemedicine users should be considered, as populations less affected by the digital divide may perceive lower risks and greater benefits compared to the groups considered disadvantaged.

### Conclusions

Context-specific consideration is useful and shows which additional factors from generally applicable frameworks or theories are particularly relevant. It also reveals which factors, if any, do not seem relevant in the context of telemedicine from an HCP’s perspective. Our results show that multidimensional transparency as an antecedent to trust, as well as the context-specific metavariables (symptom characteristics and experience), impact telemedicine adoption. The complexity and higher risk perception associated with telemedicine also lead to a multitude of perceived risks. For future research, the results of this study imply that prospective studies in the field of telemedicine should refer to the design of a modified telemedicine-contextualized valence framework to conduct a fully comprehensive analysis. The role of a broader systematic and organizational context should also be considered to understand how external factors shape telemedicine adoption. Especially when using quantitative approaches, existing scales should address telemedicine-specific characteristics of constructs such as trust referents, risks, and benefits, as well as antecedents such as transparency in their items. If they do not, the scales should be adapted accordingly.

## Appendix

Multimedia Appendix 1 Interview guide and coding schemes.

## Abbreviations

EVF: extended valence framework
HCP: health care professional
IS: information systems
TAM: technology acceptance model
UTAUT: unified theory of acceptance and use of technology

## Data Availability

The datasets generated during and analyzed during this study are available from the corresponding author on reasonable request.
