# Supplementary material for: Factors Influencing Telemedicine Adoption Among Health Care Professionals: Qualitative Interview Study
Source: JMIR Form Res. 2025 Jan 27;9:e54777. doi: 10.2196/54777 (PMC11811669; doi:10.2196/54777)
Supplement: Multimedia Appendix 1 [file formative_v9i1e54777_app1.docx]

## Appendix

Table A.1. Interview guideline.

| **Key Topic** | **Example Questions** |
| --- | --- |
| Use Behavior and Intention | Since when and how frequent do you use telemedicine? |
| Perceived Risks | What risks do you see in the use of telemedicine? |
| Perceived Benefits | What advantages does telemedicine offer compared to traditional healthcare services? |
|  | What motivates you to use telemedicine? |
| Trust Referents | Do you trust telemedicine to provide the appropriate service? |
|  | Do you think trust is relevant for the adoption of telemedicine? |
|  | In your opinion, what factors contribute to the trustworthiness of the telemedicine application? |
| Contextual Factors | Which medical applications can telemedicine be used for? |
|  | What is special and must be observed when using telemedicine? |

Table A.2. Coding Scheme for perceived risk.

| **Level 1** | **Level 2** | **Level 3** | **Definition** | **Coding rule** | **Anchor example** |
| --- | --- | --- | --- | --- | --- |
| Perceived Risk | - | - | The uncertainty and potential severity of negative consequences. *Dowling & Staelin (1994); Featherman & Pavlou (2003)* | - | - |
|  | Performance Risk | - | Performance risk reflects the perception that a product or service fails to meet the anticipated performance standards, consequently not delivering the desired benefits. *Grewal et al. (1994)* | - | - |
|  |  | *Restriction in Treatment* | The possibility of the technology restricting the treatment. | Text passages are coded in which the receiver makes a statement about Restriction in Treatment. | *The fact that I don't have the patient on site and providing treatment is evident from the name. It has to do with applying hands. I can't mechanize it. [I12]* |
|  |  | *Restriction in Patient-HCP Connection* | The possibility of the technology restricting the patient-HCP connection. | Text passages are coded in which the receiver makes a statement about Restriction in patient-HCP connection. | As I said, there are also a few potential disadvantages. The question with communication is: Do I have facial expressions and gestures in view? Like I do in real-world communication? [I5] |
|  |  | *Internet Connection Issues* | The possibility of the technology being restricted by occurring internet connection issues. | Text passages are coded in which the receiver makes a statement about internet connection issues. | It also used to happen that entire sessions got stuck. [I2] |
|  |  | *Technical Issues* | The possibility of the technology malfunctioning due to technical issues. | Text passages are coded in which the receiver makes a statement about technical issues. | It's actually often the case that conversations don't start via the platform because its technology isn't working at the moment. [I4] |
|  |  | *Technical Proficiency of Patient* | The possibility of the technology failing to deliver the desired output due to the technical proficiency of patients. | Text passages are coded in which the receiver makes a statement about technical proficiency of patient. | I think that's also the biggest issue: the technical skills of the clients. To what extent they are able to operate a laptop, install a program, check whether the microphone is working. [I4] |
|  | Financial Risk | *-* | Financial Risk reflects the potential monetary outlay due to the product’s or service’s costs. *Featherman & Pavlou (2003)* | - | - |
|  |  | *Need for Equipment* | The potential monetary outlay associated with high-tech equipment. | Text passages are coded in which the receiver makes a statement about need for equipment. | You have to invest in this program. We just bought this data-safe program. [I10] |
|  |  | *Need for Staff* | The potential monetary outlay associated with need for staff. | Text passages are coded in which the receiver makes a statement about need for staff. | There must also be physical staff to take care of a video consultation. It's not just something you do on the side. Rather, if it is to be a proper consultation, it must also be staffed. [I10] |
|  |  | *Insurance Coverage* | The potential monetary outlay associated with uncertainty about insurance coverage. | Text passages are coded in which the receiver makes a statement about insurance coverage. | Does this go towards my regular service volume from the KV practice, are these private patients that I am treating, does the provider have any contracts with health insurance companies so that the whole thing is extra-budgetary? [I13] |
|  |  | *Limited User Group* | The potential monetary outlay associated with a limited user group. | Text passages are coded in which the receiver makes a statement about limited user group. | So I think that this may not be profitable for small practices, because at the moment it is still a limited group that is suitable for this. [I7] |
|  |  | *Inadequate Compensation* | Financial loss due to additional effort which is not adequately compensated. | Text passages are coded in which the receiver makes a statement about inadequate compensation. | It is certainly a decisive factor for the use of telemedicine how it is reimbursed, if something like this is also better taken into account in the budget. [I7] |
|  | Time Risk | *-* | Time Risk reflects the potential loss of time when making a bad purchasing decision by wasting time researching and making the purchase, learning how to use a technology or service only to have to replace it if it does not perform to expectations. *Featherman & Pavlou (2003)* | - | - |
|  |  | *Familiarization with the Technology* | The potential time loss due to the familiarization with the technology. | Text passages are coded in which the receiver makes a statement about familiarization with the technology. | Once you've been trained, it's relatively easy. But it really takes a lot of time. We also did a lot of communication just among ourselves to get into the swing of things. [I6] |
|  |  | *Adaptation to the New Workflow* | The potential time loss due to the adaptation to the new workflow. | Text passages are coded in which the receiver makes a statement about adaptation to the new workflow. | However, it has to be said that a televisit or a teleconsultation with a patient in the background has a completely different workflow, a completely different work process, than just the conversation. [I6] |
|  |  | *Research for Alternatives* | The potential time loss due to the research for alternatives. | Text passages are coded in which the receiver makes a statement about research for alternatives. | So you always have to have an alternative program at hand that you can switch to quickly. [I4] |
|  | Psychological Risk | - | Psychological Risk reflects the mental stress that possibly arises through the use of technology. *Jacoby & Kaplan (1972); Featherman & Pavlou (2003)* | - | - |
|  |  | *Uncomfortable Feeling* | The risk that the use of technology causes an uncomfortable feeling. | Text passages are coded in which the receiver makes a statement about uncomfortable feeling. | Of course it was a bit exciting at the beginning. It's a bit like: If I click on it now, will I delete the Internet? [I6] |
|  |  | *Weight of Communication* | The risk that the use of technology causes an increased weight on communication. | Text passages are coded in which the receiver makes a statement about weight of communication. | You have to be aware that all the soft communication things are much more important, that this barrier via the camera is there and that you have to overcome it. [I6] |
|  |  | *External Interfering Factors* | The risk that the use of technology increases external interfering factors. | Text passages are coded in which the receiver makes a statement about external interfering factors. | I therefore believe that this risk varies from person to person and must always be considered on a case-by-case basis. [I13] |
|  | Social Risk | - | Social Risk reflects the potential loss of status in one’s social group as a result of adopting a product or service, looking foolish or untrendy. *Featherman & Pavlou (2003)* | - | - |
|  |  | *Negative Comments* | The risk of receiving negative comments because of the use of telemedicine. | Text passages are coded in which the receiver makes a statement about negative comments. | I think the social risk was perhaps even higher in 2018 and 2019 because there was a high level of skepticism and a lot of fear of contact. [I13] |
|  | Privacy Risk | - | Privacy Risk reflects the “potential loss of control over personal information”. *Featherman & Pavlou (2003)* | - | - |
|  |  | *Data Security* | The potential for not ensuring data security. | Text passages are coded in which the receiver makes a statement about data security. | The basic question is always that of data security and patient data is exchanged, so the question is: Is the server, is the transmission, is it tapped, accessed, sold on by the provider or something else? [I1] |
|  |  | *HCPs’ Privacy* | The potential for losing control of HCPs' privacy. | Text passages are coded in which the receiver makes a statement about HCPs’ privacy. | Of course, privacy must also be ensured for legal reasons. I have to be in a room where nobody else is, where nobody can hear me. [I14] |
|  |  | *Patients’ Privacy* | The potential for losing control of patients' privacy. | Text passages are coded in which the receiver makes a statement about patients’ privacy. | Compared to purely telephone-based advice, I have to say that, depending on the target group, there is of course a certain sense of shame. And certain reservations and also a certain reluctance to address very relevant topics or even quite trivial topics the first time around. [I9] |

Table A.3. Coding Scheme for perceived benefit.

| **Level 1** | **Level 2** | **Level 3** | **Definition** | **Coding rule** | **Anchor example** |
| --- | --- | --- | --- | --- | --- |
| Perceived Benefit | - | - | The potential value that a user associates with the use of telemedicine. | - | - |
|  | *Convenience of Telemedical Use* | - | The potential of telemedicine to simplify the work of HCPs. | - | - |
|  |  | *Space Independence* | The potential of telemedicine to provide space independence. | Text passages are coded in which the receiver makes a statement about space independence. | The biggest advantage is obvious. I am independent of space. [I1] |
|  |  | *Reduction of Travel Time* | The potential of telemedicine to reduce the HCP´s time of travel. | Text passages are coded in which the receiver makes a statement about his/her reduction of travel time. | No more traveling. Rehabilitation clinics, in particular, are always a little further out of town as far as our field is concerned. [I9] |
|  |  | *Time Flexibility* | The potential of telemedicine to provide flexibility with regard to timing. | Text passages are coded in which the receiver makes a statement about time flexibility. | It is also advantageous for the doctor or therapist. They can actually manage appointments more flexibly. [I8] |
|  |  | *Protection of HCP Well-Being* | The potential of telemedicine to protect HCP well-being. | Text passages are coded in which the receiver makes a statement about protection of HCP. | Of course, there will then also be less infection. [I7] |
|  |  | *Protection of Patient Well-Being* | The potential of telemedicine to protect patient well-being. | Text passages are coded in which the receiver makes a statement about protection of patient. | So if I have a cold, for example, I can do that without any problems. I've actually done this (...) I put the laptop in front of him and said: "Unfortunately, I can't see you in person, but I'd like to do the treatment and the introductory meeting this way. [I2] |
|  | *Quality of Care* | *-* | The potential of telemedicine to improve the quality of care. | - | - |
|  |  | *Accessibility* | The potential of telemedicine to increase the accessibility for patients. | Text passages are coded in which the receiver makes a statement about accessibility. | In general, you can also reach more patients, i.e. patients who are further away. [I1] |
|  |  | *Safe Space for Sensitive Topics* | The potential of telemedicine to provide a safe space for sensitive topics. | Text passages are coded in which the receiver makes a statement about safe space for sensitive topics. | I actually have the feeling that the more distance there is between medical staff and patients, whether by video or by phone, it offers patients the opportunity to mention things with a low threshold of inhibition that they might not dare to mention face to face. [I9] |
|  |  | *Enhanced Perception* | The potential of telemedicine to enhance the HCPs' perception. | Text passages are coded in which the receiver makes a statement about enhanced perception. | For me, it has proven to be more practical because it allows me to improve the performance I deliver during the session because I have less stress with things on the outside. [I3] |
|  | *Efficiency of Telemedical Service* | *-* | The potential of telemedicine to improve the work for HCPs. | - | - |
|  |  | *Time Saving* | The potential of telemedicine to save time. | Text passages are coded in which the receiver makes a statement about time saving. | If you then imagine that the patient is out and about all day, in the university hospital, in the big building, where no one really feels responsible for the patient, it is an advantage that the neurosurgery colleague has seen the patient on video and was then also able to see the images, MRI or CT images, on the computer and was then also able to speak to the patient. [I6] |
|  |  | *More Precise Working Time* | The potential of telemedicine to provide more precise working time. | Text passages are coded in which the receiver makes a statement about more precise working time. | I find more precise working hours. When I'm on site and people come and go, I have to talk to receptionists. That takes up a lot more time, including the journey to and from work. [I3] |
|  |  | *Financial Advantage* | The potential of telemedicine to provide a financial advantage. | Text passages are coded in which the receiver makes a statement about Financial Advantage. | They are not there (financial costs). We've been doing a paperless office for many years. It's just a lot of software and a lot online. It's a zero investment. [I4] |

Table A.4. Coding Scheme for trust referents.

| **Level 1** | **Level 2** | **Level 3** | **Definition** | **Coding rule** | **Anchor example** |
| --- | --- | --- | --- | --- | --- |
| Technology as Trust Referent | - | *-* |  | - | - |
|  | Technology’s Functionality | *-* | The belief that a specific technology has the appropriate functionalities or features to fulfil the requirement. *McKnight et al. (2011)* | - | - |
|  |  | *Audio Connection* | The means through which the patient and healthcare provider communicate using sound during a telemedical video consultation. | Text passages are coded in which the receiver makes a statement about Audio Connection. | If, for example, everything runs smoothly technically, i.e. the camera resolution is good, audio quality is good, everything works, end call, accept call. [I14] |
|  |  | *Visual Connection* | The video component of a telemedicine consultation that allows the patient and healthcare provider to see each other. | Text passages are coded in which the receiver makes a statement about Visual Connection. | I have the advantage that by seeing the other person or seeing them in between, it gives me more information or makes the whole thing a bit more trusting. [I1] |
|  |  | *Appointment Scheduling* | The process of arranging a specific date and time for a telemedical video consultation. | Text passages are coded in which the receiver makes a statement about Appointment Scheduling. | If the appointment is also scheduled where you have scheduled it, this simply leads to a smooth process and thus also strengthens trust in the technology or in the method itself. [I14] |
|  |  | *Anamnesis Data Take* | The collection and recording of a patient's medical history and current symptoms during a telemedicine consultation. | Text passages are coded in which the receiver makes a statement about Anamnesis Data Take. | And you can often have photos sent to you in advance. [I13] |
|  | Technology’s Reliability | *-* | The belief that the specific technology will consistently operate properly. *McKnight et al. (2011)* | - | - |
|  |  | *Stable Connection* | A reliable and uninterrupted internet service that is crucial for the smooth and effective communication during a telemedical video consultation. | Text passages are coded in which the receiver makes a statement about Stable Connection. | If we now have a Zoom call and we have half an hour until we can understand each other, then trust naturally drops. [I1] |
|  |  | *Stable Platform* | A dependable and consistent telemedicine software or application that supports video consultations without technical disruptions. | Text passages are coded in which the receiver makes a statement about Stable Platform. | (…) then the server of the specific provider cannot be reached, (…) [I3] |
| Treatment as Trust Referent | *-* | *-* |  | - | - |
|  | *1. Consensus Understanding of Treatment Process* | *-* |  | - | - |
|  |  | *Practicality* | The ease and convenience of conducting a telemedical video consultation for both the patient and HCP. | Text passages are coded in which the receiver makes a statement about Practicality. | What matters most in actual practice is something more like practicality. [I1] |
|  |  | *Enabling Adequate Dialog* | Creating an environment during a telemedical video consultation that supports effective communication between the patient and HCP. | Text passages are coded in which the receiver makes a statement about Enabling Adequate Dialog. | Does the doctor have the basic capability to communicate with confidence and to communicate components in such a way that they are well received by the other side and are also seen as useful? [I2] |
|  | *2. Effectiveness of Collaboration* | *-* |  | - | - |
|  |  | *Consent of Patient* | The patient's agreement to participate in a telemedical video consultation after being informed about its nature and implications. | Text passages are coded in which the receiver makes a statement about Consent of Patient. | We also obtain consent from each patient as part of the data protection declaration. [I11] |
|  |  | *Enabling Compliance* | Facilitating the patient's adherence to medical advice or treatment plans during and after a telemedical video consultation. | Text passages are coded in which the receiver makes a statement about Enabling Compliance. | I always consider the initial contact to be the most important contact of all. We can make it clear there: What is the service that can be provided now? [I9] |
|  |  | *HCP-Patient Connection* | The establishment of a rapport and trustful relationship between the patient and HCP during a telemedical video consultation. | Text passages are coded in which the receiver makes a statement about HCP-Patient Connection. | If they trust me here in the clinic now, I don't see why they shouldn't just trust me in front of the camera, because they already knew me beforehand. [I6] |
|  |  | *Patient Feedback* | The feedback provided by the patient regarding their experience and outcomes of the telemedical video consultation. | Text passages are coded in which the receiver makes a statement about Patient Feedback. | The patients I did it with gave me positive feedback because they liked it. And if it works and you don't have any negative experiences, plus the reinforcement from the positive feedback from the patients, yes, that encourages you to go ahead and do it. [I7] |
|  | *3. Effectiveness of Treatment* | *-* |  | - | - |
|  |  | *Provision of Medical Reports* | The effective process of delivering medical findings, diagnoses, or treatment plans to the patient during or after a telemedical video consultation. | Text passages are coded in which the receiver makes a statement about Provision of Medical Reports. | I can share my screen in the other system. You can watch or view files together. [I6] |
|  |  | *Work Facilitation* | The enhancement of effectiveness for healthcare providers in delivering care through telemedical video consultations. | Text passages are coded in which the receiver makes a statement about Work Facilitation. | I find it trustworthy because it simply makes my work easier and also expands my work and gives me new opportunities. [I11] |
|  |  | *Possible Guidance to Self-Examination* | Effectiveness of instructions provided by the healthcare provider to the patient for conducting self-assessment or examination during a telemedical video consultation. | Text passages are coded in which the receiver makes a statement about Possible Guidance to Self-Examination. | Then I think it's often enough to advise the patient accordingly or, let's say, to instruct them in small self-examinations. [I13] |
| Provider as Trust Referent | - | *-* |  | - | - |
|  | Provider Ability | *-* | The skills, competencies, and attributes that enable the provider to have an impact in a specific domain. *Mayer et al. (1995)* | - | - |
|  |  | *Contextual Knowledge* | The provider's understanding of the telemedical process. | Text passages are coded in which the receiver makes a statement about Contextual Knowledge. | Yes, it is (trustworthy). Provided that you don't do it with Zoom or WebEx or FaceTime or Whatsapp, but that you use a platform that is intended for this purpose by the German Medical Association and also meets the relevant criteria. |
|  |  | *Technical Knowledge* | The provider's expertise on technology. | Text passages are coded in which the receiver makes a statement about Technical Knowledge. | I don't have that much trust in the technology, but because I have a lot of choice, I can change providers quickly if I know my way around. [I3] |
|  | Provider Benevolence | *-* | The degree to which the provider intends goodwill toward the HCP, alongside their perceived positive attitude towards the HCP. *Mayer et al. (1995)* |  |  |
|  |  | *Proximity* | The sense of closeness or personal attention a provider can convey. | Text passages are coded in which the receiver makes a statement about Proximity. | Jameda is a German company, if I remember correctly even a Munich-based company. This means that you somehow have a localized trust building. [I1] |
|  | Provider Integrity | *-* | The HCP’s perception that the provider adheres to a set of principles that the HCP finds acceptable. *Mayer et al. (1995)* | - | - |
|  |  | *Data Security* | The provider's commitment and capability to protect patient information, ensuring confidentiality. | Text passages are coded in which the receiver makes a statement about Data Security. | And one question that was important to me was: where is the data storage, the data backup (...) I think that's a very important factor, basically what builds trust. [I1] |
|  |  | *Responsiveness* | The provider's ability to promptly and adequately respond to inquiries or concerns. | Text passages are coded in which the receiver makes a statement about Responsiveness. | Whenever we had difficulties, we always received support quickly. [I10] |
|  |  | *Certification* | The provider's possession of recognized qualifications or credentials. | Text passages are coded in which the receiver makes a statement about Certification. | Certification either as a medical device or as a clear video conference by a recognized certification body would be an important issue. [I5] |
| Patient as Trust Referent | - | *-* |  | - | - |
|  | Patient Ability | *-* | The skills, competencies, and attributes that enable the patient to have an impact in a specific domain. *Mayer et al. (1995)* | - | - |
|  |  | *Digital Affinity* | The patient's comfort and familiarity with using digital technology, contributing to their trust and ease in engaging with telemedical services. | Text passages are coded in which the receiver makes a statement about Digital Affinity. | The limiting factor mentioned is sometimes the technical understanding of clients or patients. [I4] |

Table A.5. Coding Scheme for transparency.

| **Level 1** | **Level 2** | **Level 3** | **Definition** | **Coding rule** | **Anchor example** |
| --- | --- | --- | --- | --- | --- |
| Transparency | - | - | The perceived quality of intentionally shared information from a sender. *Schnackenberg & Tomlinson (2016)* | - | - |
|  | Transparency on Technology | - | The perceived quality of intentionally shared information on Technology from a sender. *Schnackenberg & Tomlinson (2016)* | - | - |
|  |  | Disclosure | The belief that all relevant information on technology is shared in a timely manner. | Text passages are coded in which the receiver makes a statement about Disclosure on Technology. | Yes, although the question when using it as a medical tool in patient communication is: What is allowed? What is problematic in terms of data protection law? If not, are there certificates? [I5] |
|  |  | Clarity | The level of correspondence between the intended and the understood meaning of information on technology. | Text passages are coded in which the receiver makes a statement about Clarity on Technology. |  |
|  |  | Accuracy | The extent to which information on technology given matches reality and is free from intentional distortions. | Text passages are coded in which the receiver makes a statement about Accuracy on Technology. | And since I've read up on it and mainly use a provider in Germany, I already know what he gets. I'm not a technician and can't estimate what he could do with this data, but I feel well informed. [I3] |
|  | Transparency on Treatment | - | The perceived quality of intentionally shared information on the telemedical treatment from a sender. *Schnackenberg & Tomlinson (2016)* | - | - |
|  |  | Disclosure | The belief that all relevant information on treatment is shared in a timely manner. | Text passages are coded in which the receiver makes a statement about Disclosure on Treatment. | You can read everything in the terms and conditions or data protection agreements if something is missing. [I9] |
|  |  | Clarity | The level of correspondence between the intended and the understood meaning of information on treatment. | Text passages are coded in which the receiver makes a statement about Clarity on Treatment. | There is certainly still a lack of information flow and clarity (on telemedicine). [I5] |
|  |  | Accuracy | The extent to which information on treatment given matches reality and is free from intentional distortions. | Text passages are coded in which the receiver makes a statement about Accuracy on Treatment. | I am obliged to do this (inform on treatment) before I enter into a patient contract. [I11] |
|  | Transparency on Provider | - | The perceived quality of intentionally shared information on the provider from a sender. *Schnackenberg & Tomlinson (2016)* | - | - |
|  |  | Disclosure | The belief that all relevant information on provider is shared in a timely manner. | Text passages are coded in which the receiver makes a statement about Disclosure on Provider. | It's good to show what kind of partner, software partner, am I working with? Is it one that patients can google in advance and find something about and perhaps see that they have certification and are data protection-certified? [I13] |
|  |  | Clarity | The level of correspondence between the intended and the understood meaning of information on provider. | Text passages are coded in which the receiver makes a statement about Clarity on Provider. | Clarity, do you mean clarity of procedures? (...) Yes, yes, definitely. [I11] |
|  |  | Accuracy | The extent to which information on provider given matches reality and is free from intentional distortions. | Text passages are coded in which the receiver makes a statement about Accuracy on Provider. | That always varies depending on the provider. Some are very open, very transparent. They provide a lot of information, answer all your questions and you always have the feeling that you're being shown everything transparently. [I13] |

Table A.6. Coding Scheme for context variables.

| **Level 1** | **Level 2** | **Definition** | **Coding rule** | **Anchor example** |
| --- | --- | --- | --- | --- |
| Context variable | - |  | - | - |
|  | Symptom Characteristics | Specific aspects and details of a patient's symptoms that determine whether telemedicine is a sensible approach. | Text passages are coded in which the receiver makes a statement about Symptom Characteristics. | It is not an option for everyone. Not only because of the disease, but also because of the disease. [I7] |
|  | Experience | The accumulated knowledge and skills of the HCP in managing and treating patients in the setting of telemedicine. | Text passages are coded in which the receiver makes a statement about Experience. | I would describe it as very good. Surprisingly good. I've been using it since Corona, starting with the first wave of Corona, and I now have every fifth consultation online. [I2] |

## References

Dowling GR, Staelin R. A Model of Perceived Risk and Intended Risk-Handling Activity. Journal of Consumer Research 1994;21(1):119-134. doi:10.1086/209386

Featherman M, Pavlou PA. Predicting e-services adoption: a perceived risk facets perspective. International Journal of Human-Computer Studies 2003;59(4):451-474. doi:10.1016/S1071-5819(03)00111-3

Grewal D, Gotlieb J, Marmorstein H. The moderating effects of message framing and source credibility on the price-perceived risk relationship. Journal of Consumer Research 1994;21(1):145-153. doi:10.1086/209388

Jacoby J, Kaplan LB. The Components of Perceived Risk. In: Venkatesan M, editor. Proceedings of the Third Annual Conference of the Association for Consumer Research; 1972. p. 382–393.

Mayer RC, Davis JH, Schoorman FD. An Integrative Model of Organizational Trust. Academy of Management Review 1995;20(3):709-734. doi:10.2307/258792

McKnight DH, Carter M, Thatcher JB, Clay PF. Trust in a specific technology: An investigation of its components and measures. ACM Transactions on Management Information Systems 2011;2(2):12-32.

Schnackenberg AK, Tomlinson EC. Organizational Transparency. Journal of Management 2016;42(7):1784-1810. doi:10.1177/0149206314525202
